# Mixing of quantum states: A new route to creating optical activity

**DOI:** 10.1038/s41598-016-0017-0

**Published:** 2016-12-05

**Authors:** Anvar S. Baimuratov, Nikita V. Tepliakov, Yurii K. Gun’ko, Alexander V. Baranov, Anatoly V. Fedorov, Ivan D. Rukhlenko

**Affiliations:** 1 ITMO University, 197101 Saint Petersburg, Russia; 2 School of Chemistry and CRANN Institute, Trinity College, Dublin, Dublin 2 Ireland; 3 Monash University, Clayton Campus, Victoria, 3800 Australia

## Abstract

The ability to induce optical activity in nanoparticles and dynamically control its strength is of great practical importance due to potential applications in various areas, including biochemistry, toxicology, and pharmaceutical science. Here we propose a new method of creating optical activity in originally achiral quantum nanostructures based on the mixing of their energy states of different parities. The mixing can be achieved by selective excitation of specific states or *via* perturbing all the states in a controllable fashion. We analyze the general features of the so produced optical activity and elucidate the conditions required to realize the total dissymmetry of optical response. The proposed approach is applicable to a broad variety of real systems that can be used to advance chiroptical devices and methods.

## Introduction

Chirality is known to occur at many levels of life organization and plays a key role in many chemical and biological processes in nature^1^. The fact that most of organic molecules are chiral starts more and more affecting the progress of contemporary medicine and pharmaceutical industry^2^. As a consequence, a great deal of research efforts has been recently focused on the optical activity of inherently chiral organic molecules^3, 4^. It can be strong in the ultraviolet range, in which case it is difficult to study and use in practice. Shifting the chiroptical response of such molecules into the visible domain is quite challenging and can be achieved, for example, by coupling chiral molecules or biomolecules to plasmonic nanoparticles^5–9^.

With the advent of the nanotechnology era it has become possible to create and engineer optical activity in various kinds of inorganic nanostructures^10^. There are several approaches to making a nanostructure optically active. One way of doing this is to order achiral nanoparticles or nanocrystals into chiral structures, such as helixes or tetrahedra^9, 11, 12^. Another way is the coupling of an achiral nanoparticle to chiral molecules that can transfer their enantiomeric structure to the nanoparticle surface^13–15^. Besides this, one can fabricate nanoparticles of chiral shapes^16, 17^ or with screw dislocations of the crystal lattice^18–22^. It should be noted that the induction of optical activity in all these cases requires nanostructures not only to lack the mirror symmetry, but also to preserve the helicity of light^23, 24^. Most importantly is that the ability to control the size, geometry, and composition of chiral nanostructures enables the tunability of their optical response over a broad range of electromagnetic spectrum, including ultraviolet, visible, and infrared ranges. Studying the optical activity of nanostructures is thus of applied significance and has a potential to produce new materials and devices with controllable optical properties.

Despite the variety of ways to achieve optical activity in inorganic nanostructures, they all are underlaid by the same physical principle, which is the breaking of mirror symmetry of the nanostructure’s electronic subsystem. This raises a number of fundamental questions, such as (i) what are the general conditions to be satisfied for a nanostructure to exhibit optical activity, (ii) how can this activity be maximized for a given set of material or geometric parameters, and (iii) whether or not the total dissymmetry of optical response of nanostructures is feasible. With this paper we offer an approach to address these questions using general considerations and without specifying the exact physical origin of the nanostructure’s chirality. This approach leads us to a number of valuable results and a simple theoretical formalism that can prove useful in predicting and interpreting experimental data.

## Results

Let us consider the circumstance in which an arbitrary quantum mechanical system with a discrete energy spectrum exhibits optical activity, that is displays different interactions with left circular polarized (LCP) light and right circular polarized (RCP) light. The measure of optical activity upon the system’s transition from one state to another is the difference between the absorption rates of LCP and RCP light upon this transition, known as the *rotatory strength*. For quantum systems such as small molecules and semiconductor nanocrystals, whose dimensions are much smaller than the optical wavelength, the rotatory strength is given by Rosenfeld’s formula^25^

1 $${R}_{fi}={\rm{Im}}(\langle i|{\bf{d}}|\,f\rangle \langle \,f|{\bf{m}}|i\rangle ),$$

where |*i*〉 and | *f* 〉 are the initial and final states of the transition, and **d** and **m** are the electric and magnetic dipole moments. The strength of enantioselectivity in the interaction of a quantum system with circularly polarized light is characterized by the *dissymmetry factor*, which is the ratio of the rotatory strength to the full dipole absorption rate^26^,

2 $${g}_{fi}=\frac{4\,{\rm{Im}}(\langle i|{\bf{d}}|\,f\rangle \langle \,f|{\bf{m}}|i\rangle )}{{|\langle i|{\bf{d}}|f\rangle |}^{2}+{|\langle f|{\bf{m}}|i\rangle |}^{2}}.$$

One can see that transition |*i*〉 → |* f* 〉 is optically active, provided matrix elements 〈*i*|**d**| *f* 〉 and 〈 *f*|**m**|*i* 〉 are both nonzero and not mutually orthogonal. The first condition implies that the considered transition should be simultaneously electric dipole and magnetic dipole allowed whereas the second one requires the two kinds of transitions to have the same selection rules for the respective components of **d** and **m**. Note that the identity of the selection rules is necessary but insufficient, because the scalar product can vanish even in case of individual vector components being all nonzero.

The above definition of rotatory strength admits a generic method of creating optical activity in a quantum system whose eigenstates are originally achiral. Suppose that the system can transition from an initial state |*i*〉 to a superposition of achiral eigenstates |*a*〉 and |*b*〉 of different parities, such that transition |*i*〉 → |*a*〉 is electric dipole allowed and transition |*i*〉 → |*b*〉 is magnetic dipole allowed. Owing to the different parities of states |*a*〉 and |*b*〉, their mixture |* f*〉 = *c*_*a*_|*a*〉 + *c*_*b*_|*b*〉 is chiral in the sense that its wave function 〈**r**| *f*〉 does not have a mirror symmetry plane. In most instances, the matrix element of the electric dipole moment is real and the matrix element of the magnetic dipole moment is pure imaginary^27^, so that we can take 〈*i*|**d**|*a*〉 = **d**_*ia*_ and 〈*b*|**m**|*i*〉 = *i* **m**_*bi*_, where **d**_*ia*_ and **m**_*bi*_ are the real vectors, to obtain

3 $${R}_{fi}=({{\bf{d}}}_{ia}\cdot {{\bf{m}}}_{bi}){\rm{Re}}({c}_{a}{c}_{b}^{\ast }),$$

4 $${g}_{fi}=\frac{4{R}_{fi}}{{|{c}_{a}|}^{2}{{\bf{d}}}_{ia}^{2}+{|{c}_{b}|}^{2}{{\bf{m}}}_{bi}^{2}}.$$

These expressions show that the rotatory strength and dissymmetry factor are maximal when **d**_*ia*_ is parallel to **m**_*bi*_. Furthermore, since coefficients *c*_*a*_ and *c*_*b*_ are bound by the normalization condition |*c*_*a*_|^2^ + |*c*_*b*_|^2^ = 1, the maximum of *R*_*fi*_ is attained for *c*_*a*_ = ±*c*_*b*_ and $|{c}_{a}|=1/\sqrt{2}$. It is also seen that *g*_*fi*_ reaches its maximal value when *c*_*a*_ **d**_*ia*_ = ±*c*_*b*_ **m**_*bi*_. The possibility to satisfy this condition by adjusting the weights of the quantum states in the mixture — needed since $|{{\bf{d}}}_{ia}|\gg |{{\bf{m}}}_{bi}|$ in typical quantum systems like semiconductor quantum dots — paves a way to the realization of a complete dissymmetry of dipole absorption.

## Discussion

One of the possible implementations of the proposed generic method is the following. Suppose that quantum states |*a*〉 and |*b*〉 of close energies *E*_*a*_ and *E*_*b*_ = *E*_*a*_ − Δ*E* are coupled by some interaction, whose strength is characterized by the matrix element $\langle b|\hat{V}|a\rangle =V{e}^{i\phi }$. By coupling the states of different parities, this interaction introduces chirality to the originally achiral quantum system. The coupling can be induced by screw dislocations, impurity ions, point defects, imperfections of the nanocrystal shape, magnetic field, *etc.* ^20, 21, 28–31^. The stationary perturbation theory predicts that the coupled states hybridize, resulting in the formation of new states $|{f}^{(\pm )}\rangle ={c}_{a}^{(\pm )}|a\rangle +{c}_{b}^{(\pm )}|b\rangle$ of energies ${E}_{f}^{(\pm )}=({E}_{a}+{E}_{b}\pm \beta )/2$, with ${c}_{a}^{(\pm )}={e}^{-i\phi /2}\sqrt{(1\pm {\rm{\Delta }}E/\beta )/2}$, ${c}_{b}^{(\pm )}=\pm {e}^{i\phi /2}\sqrt{(1\mp {\rm{\Delta }}E/\beta )/2}$, and $\beta =\sqrt{{\rm{\Delta }}{E}^{2}+4{V}^{2}}$ ^32^. One can see that the weights of states |*a*〉 and |*b*〉 are controlled by the ratio of their energy detuning Δ*E* to the interaction strength *V*. The rotatory strengths of optical transitions to the hybrid states are given by

5 $${R}_{fi}^{(\pm )}=\pm ({{\bf{d}}}_{ia}\cdot {{\bf{m}}}_{bi})(V/\beta )\,\cos \,\varphi .$$

These strengths peak at the exact resonance between the coupled states (Δ*E* = 0) whereas the respective dissymmetry factors both peak at $\pm {\rm{\Delta }}E/V=|{{\bf{d}}}_{ia}|/|{{\bf{m}}}_{bi}|-|{{\bf{m}}}_{bi}|/|{{\bf{d}}}_{ia}|\approx |{{\bf{d}}}_{ia}|/|{{\bf{m}}}_{bi}|\gg 1$, which implies that strong dissymmetry of optical response results in weak optical activity and *vice versa*. Since the weights of resonant states in the mixture are equal, the rotatory strengths in this case do not depend on *V*. If ratio Δ*E*/*V* is optimal, ${{\bf{d}}}_{ia}\parallel {{\bf{m}}}_{bi}$, and *φ* = *πn* (*n* = 0, ±1, ±2, …), then the two dissymmetry factors can reach their highest possible values of ±2. These values imply that upon each of the transitions the system fully absorbs light of one circular polarization and does not absorb light of the other.

The optical activity of a quantum system shows up in its circular dichroism (CD) spectrum^27^. The proposed hybridization mechanism leads to a specific shape of this spectrum, which can be represented in the form ${\rm{CD}}(\omega )\propto {R}_{fi}^{(+)}{\rm{\Gamma }}({E}_{f}^{(+)},\omega )+{R}_{fi}^{(-)}{\rm{\Gamma }}({E}_{f}^{(-)},\omega )$, where Γ(*E*, *ω*) is the spectral lineshape centered at frequency *E*/*ħ*. Using Eq. (4) and approximating the lineshapes of transitions |*i*〉 → |* f* ^(±)^〉 by Lorentzians of the same full width at half maximum (FWHM) 2*γ*, we find that our model leads to the spectrum

6 $${\rm{CD}}({\rm{\Delta }}E,V,\gamma ,\delta )\propto \frac{2\gamma V\delta }{[(\delta +\beta /{\mathrm{2)}}^{2}+{\gamma }^{2}][{(\delta -\beta /\mathrm{2)}}^{2}+{\gamma }^{2}]},$$

where *δ* = *ħω* − (*E*_*a*_ + *E*_*b*_)/2 and we have assumed that the energy of initial state is zero.

**Figure 1:**
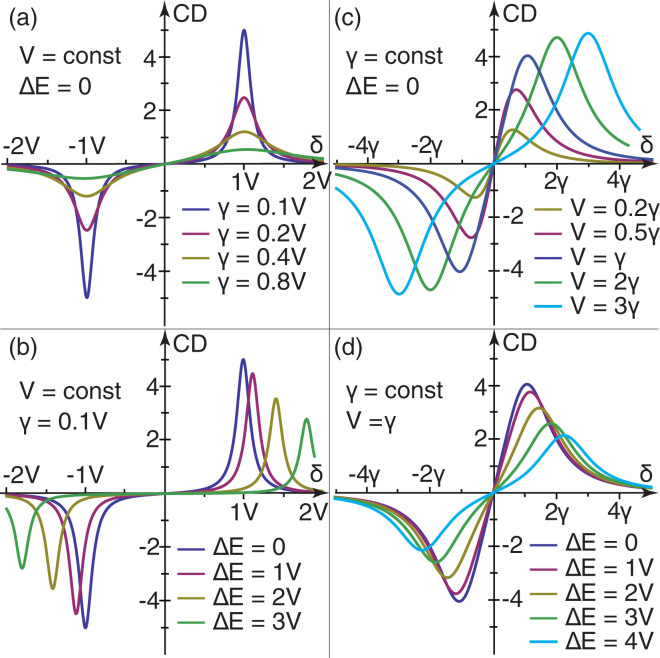
CD spectra (in arbitrary units) peculiar to optically active systems with two quantum states coupled by an interaction of strength V. The spectral lineshapes are approximated by Lorentzians of FWHMs 2*γ*. The upper and lower panels represent resonant and off-resonant spectra, respectively.

Figure 1 shows the CD spectrum given by Eq. (5) as a function of energy detuning *δ* for fixed interaction strength [panels (a) and (b)] and for fixed spectral linewidths [panels (c) and (d)]. Several general features of optical activity induced by mixing of quantum states are evidenced by this figure. First of all, the CD peaks representing transitions to the mixed states are equally strong and the most pronounced at the exact resonance between the coupled stated. If the peaks are relatively narrow, $\gamma \ll \beta$, their FWHMs equal 2*γ* and their positions approximately coincide with the transition energies *δ*_±_ = ±*β*/2. An increase of the linewidth weakens the peaks and makes them drift apart. The positions of the peaks in the CD spectrum are given by

7 $${\delta }_{\pm }=\pm \frac{1}{2\sqrt{3}}\sqrt{{\beta }^{2}-4{\gamma }^{2}+2\sqrt{{\beta }^{4}+4{\beta }^{2}{\gamma }^{2}+16{\gamma }^{4}}}.$$

In case of broad peaks, with $\gamma \gg \beta$, from this expression and Eq. (5) we find that the separation and FWHMs of the peaks can be approximated as ${\delta }_{+}-{\delta }_{-}\approx 2\gamma /\sqrt{3}$ and 1.2*γ*.

**Figure 2:**
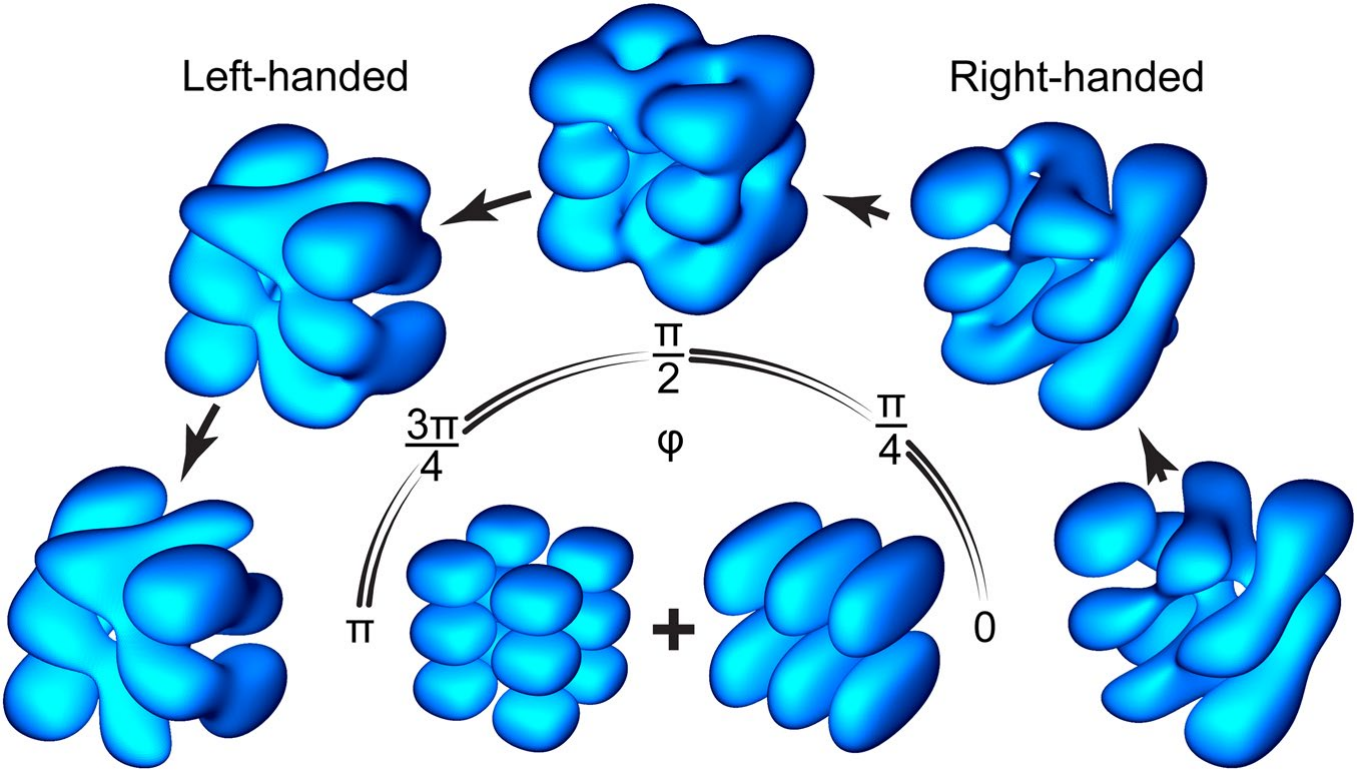
Transformation of probability density |〈r| f^ (+)^〉|^2^ with phase φ of interaction coupling a pair of resonant nanocuboid states |223〉 and |312〉.

Equation (4) shows that the rotatory strengths critically depend on the phase of the coupling interaction, varying like $\propto \,\cos \,\phi$. This feature is illustrated in Fig. 2 by the example of resonant electronic states |*a*〉 = |223〉 and |*b*〉 = |312〉 coupled inside a semiconductor nanocuboid with an impenetrable surface. The probability density of the mixed state $|\,{f}^{(+)}\rangle =({e}^{i\phi \mathrm{/2}}|a\rangle +{e}^{-i\phi \mathrm{/2}}|b\rangle )/\sqrt{2}$ is seen to exhibit chirality, the extent of which gradually reduces to zero as *φ* is increased from zero to *π*/2, and then starts growing as *φ* is increased further toward *π*.

It should be noted that the total dissymmetry of dipole absorption can be achieved even in the situation of exact resonance between the mixed states, in which ${c}_{a}^{(\pm )}=\pm {c}_{b}^{(\pm )}$. This can be done by coupling quantum states of the same or different nanoparticles with **d**_*ia*_ = ±**m**_*bi*_. Consider, for example, a pair of resonant electronic states $|a\rangle =|{a}_{x},{a}_{y},{a}_{z}\rangle$ and $|b\rangle =|{b}_{x},{b}_{y},{b}_{z}\rangle$ $({a}_{\nu },{b}_{\nu }=1,2,3,\ldots )$ of a semiconductor nanocuboid *L*_*x*_ × *L*_*y*_ × *L*_*z*_, which provides an infinite potential barrier for its confined electrons^33, 34^. Let us focus on the electric dipole absorption associated with the confined motion along the *x* axis, and on the magnetic dipole absorption associated with the confined motion in the *yz* plane, in which case |*i*〉 = |*b*_*x*_, *a*_*y*_, *a*_*z*_〉. Then by taking real wave functions and equating the two matrix elements, we obtain

8 $$2c\langle {a}_{x}|x|{b}_{x}\rangle =\sqrt{\varepsilon }({\omega }_{{b}_{z}{a}_{z}}-{\omega }_{{b}_{y}{a}_{y}})\langle {a}_{y}|y|{b}_{y}\rangle \langle {a}_{z}|z|{b}_{z}\rangle ,$$

where *c* is the speed of light in vacuum, *ε* is the high-frequency permittivity of the nanocuboid, ${\omega }_{\alpha \beta }=({E}_{\alpha }-{E}_{\beta })/\hslash$, ${E}_{\alpha }={(\pi \hslash \alpha )}^{2}/(2m{L}_{\alpha }^{2})$, *m* is the effective mass of electrons, and $\langle {a}_{\nu }|\nu |{b}_{\nu }\rangle =({L}_{x}/{\pi }^{2})$ $\,8{a}_{\nu }{b}_{\nu }/{({a}_{\nu }^{2}-{b}_{\nu }^{2})}^{2}\,{\rm{Re}}({i}^{{a}_{\nu }+{b}_{\nu }+1})$. Together with the resonance condition ${E}_{{a}_{x}}+{E}_{{a}_{y}}+{E}_{{a}_{z}}$ $={E}_{{b}_{x}}+{E}_{{b}_{y}}+{E}_{{b}_{z}}$, Eq. (7) determines the relationship between the nanocuboid’s dimensions and the quantum numbers of the coupled states that result in the total dissymmetry of optical response. For example, the pair of states in Fig. 2 results in ${L}_{x}\approx 40{\lambda }_{c}$, ${L}_{y}\approx 31{\lambda }_{c}$, and ${L}_{z}\approx 560{\lambda }_{c}$, where ${\lambda }_{c}=\sqrt{\varepsilon }\,\hslash /(mc)$ is the effective Compton wavelength. Without loss of generality we assume the typical material parameters of a bulk semiconductor by taking *m* ~ 0.03*m*_0_ (*m*_0_ is the free-electron mass) and *ε* ~ 10, we get ${\lambda }_{c}\approx 0.41\,\AA$. One can see that the realization of the total dissymmetry of dipole absorption in the case of Δ*E* = 0 requires an extremely fine tuning of the nanocrystal dimensions.

As a final illustration of the suggested approach we consider a resonance between quantum states of electron–hole pairs in two semiconductor nanorods. The matrix elements of the electric-dipole and magnetic-dipole interband transitions inside the nanorods are given by^35^

9 $${{\bf{d}}}_{ia}=-e{{\bf{P}}}_{cv},\,{{\bf{m}}}_{bi}=-\pi \sqrt{\varepsilon }(e/\lambda )\,{{\bf{r}}}_{bi}\times {{\bf{P}}}_{cv},$$

where **P**_*cv*_ and **r**_*bi*_ are the matrix elements of the radius vector calculated using Bloch functions and envelope wave functions, respectively, *e* is the elementary charge, and *λ* is the excitation wavelength in vacuum. Suppose that in each of the nanorods **P**_*cv*_ is parallel to the nanorod’s axis^36^, and denote this vector in the nanorods as ${{\bf{P}}}_{cv}^{{\rm{A}}}$ and ${{\bf{P}}}_{cv}^{{\rm{B}}}$. One can always make the electric dipole moment of A parallel to the magnetic dipole moment of B by adjusting the mutual orientation of the nanorods. Similar to the case of a chiral dimer^37^ and coupled quantum oscillators^38^, this requires positioning the nanorods in such a way that ${{\bf{P}}}_{cv}^{{\rm{A}}}\perp {{\bf{P}}}_{cv}^{{\rm{B}}}$. The maximal rotatory strength and dissymmetry factor for Δ*E* = 0 and ${c}_{a}^{(\pm )}=\pm {c}_{b}^{(\pm )}$ are then given by

10 $$|{R}_{fi}^{\pm }|=\frac{1}{2}|{{\bf{d}}}_{ia}^{{\rm{A}}}||{{\bf{m}}}_{bi}^{{\rm{B}}}|=\frac{\pi }{2}\sqrt{\varepsilon }\,{e}^{2}|{{\bf{P}}}_{cv}^{{\rm{A}}}||{{\bf{P}}}_{cv}^{{\rm{B}}}|\frac{r}{\lambda },$$

11 $$|{g}_{fi}^{\pm }|\approx 8|{R}_{fi}^{\pm }|/{({{\bf{d}}}_{ia}^{{\rm{A}}})}^{2}=4\pi \sqrt{\varepsilon }r/\lambda ,$$

where $r={({{\bf{r}}}_{bi}^{{\rm{B}}})}_{\perp }$ is the component of **r**_*bi*_ perpendicular to the plane formed by vectors ${{\bf{P}}}_{cv}^{{\rm{A}}}$ and ${{\bf{P}}}_{cv}^{{\rm{B}}}$, and we have assumed that $|{{\bf{d}}}_{ia}^{{\rm{A}}}|\gg |{{\bf{m}}}_{bi}^{{\rm{B}}}|$. The result to note here is that the absolute and relative strengths of optical activity scale as the ratio of the nanorod B’s transverse dimension *r* ~ *L* and wavelength *λ*.

**Figure 3:**
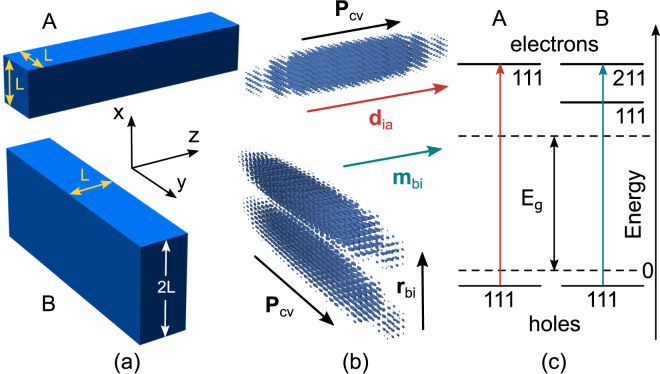
Schematic of (a) nanorods A and B, (b) wave functions of electronic states |111〉 and |211〉, electric and magnetic dipole moments of interband transitions, and (c) energy-level diagrams of the considered transitions; *E*_g_ is the bandgap of bulk material.

Figure 3 shows a possible realization of the described scenario. Nanorods A and B are aligned in the *z* and *y* directions, so that only the *x* component of the radius vector contributes to the matrix element *r*. The mutual arrangements of the nanorods and vectors ${{\bf{d}}}_{ia}^{{\rm{A}}}$, ${{\bf{m}}}_{bi}^{{\rm{B}}}$, ${{\bf{P}}}_{cv}^{{\rm{A}}}$, ${{\bf{P}}}_{cv}^{{\rm{B}}}$, and ${{\bf{r}}}_{bi}^{{\rm{B}}}$ inside them are shown in Fig. 3(a,b). Let the optical excitation lead to electric dipole transition |111〉_*h*_ → |111〉_*e*_ between the states of holes (*h*) and electrons (*e*) and to magnetic dipole transition |111〉_*h*_ → |211〉_*e*_ shown in Fig. 3(c). If the effective mass of holes is much larger than the effective mass of electrons, then the resonance between electron–hole pair states |*a*〉_A_ = |111; 111〉_A_ and |*b*〉_B_ = |211; 111〉_B_ (in which the first three quantum numbers describe electrons and the last three — holes) takes place in the nanorods of dimensions *L* × *L* × *L*_*z*_ and 2*L* × *L* × *L*_*z*_, with ${L}_{z}\gg L$. The optical activity upon transitions from the electron–hole pair vacuum to the mixtures of states |*a*〉_A_ and |*b*〉_B_ is then characterized by Eq. (9) with $r=\langle 2|x|1\rangle ={[4/(3\pi )]}^{2}L$.

In conclusion, we have proposed a new generic method of making arbitrary quantum mechanical systems optically active, which relies on the mixing of quantum states of different parities. This mixing introduces chirality into the system and can be achieved by selective excitation of energy states or *via* coupling the states by perturbing the system. We analyzed the general features of the so created optically active systems, with an emphasis on the maximization of their chiroptical response. It was also shown how to make a chiral system with the mixed quantum states which fully absorbs light of one circular polarization and does not absorb light of the other. Being applicable to a large class of real systems, our results are of potential importance for applications in relevant areas of biology, chemistry, and medicine.
