# Erratum: Mixing of quantum states: A new route to creating optical activity

**DOI:** 10.1038/s41598-017-00064-4

**Published:** 2017-06-01

**Authors:** Anvar S. Baimuratov, Nikita V. Tepliakov, Yurii K. Gun’ko, Alexander V. Baranov, Anatoly V. Fedorov, Ivan D. Rukhlenko

**Affiliations:** 1 ITMO University, 197101 Saint Petersburg, Russia; 2 School of Chemistry and CRANN Institute, Trinity College, Dublin, Dublin 2 Ireland; 3 Monash University, Clayton Campus, Victoria, 3800 Australia

## Abstract

A correction to this article has been published and is linked from the HTML version of this paper. The error has not been fixed in the paper.

*Scientific Reports* **6**:5; doi:10.1038/s41598-016-0017-0; Article published online 05 December 2016

This Article contains an incorrect version of Figure 2, where ‘π’ was incorrectly displayed as ‘e’. The correct Figure 2 appears below as Figure 1.

**Figure 1:**
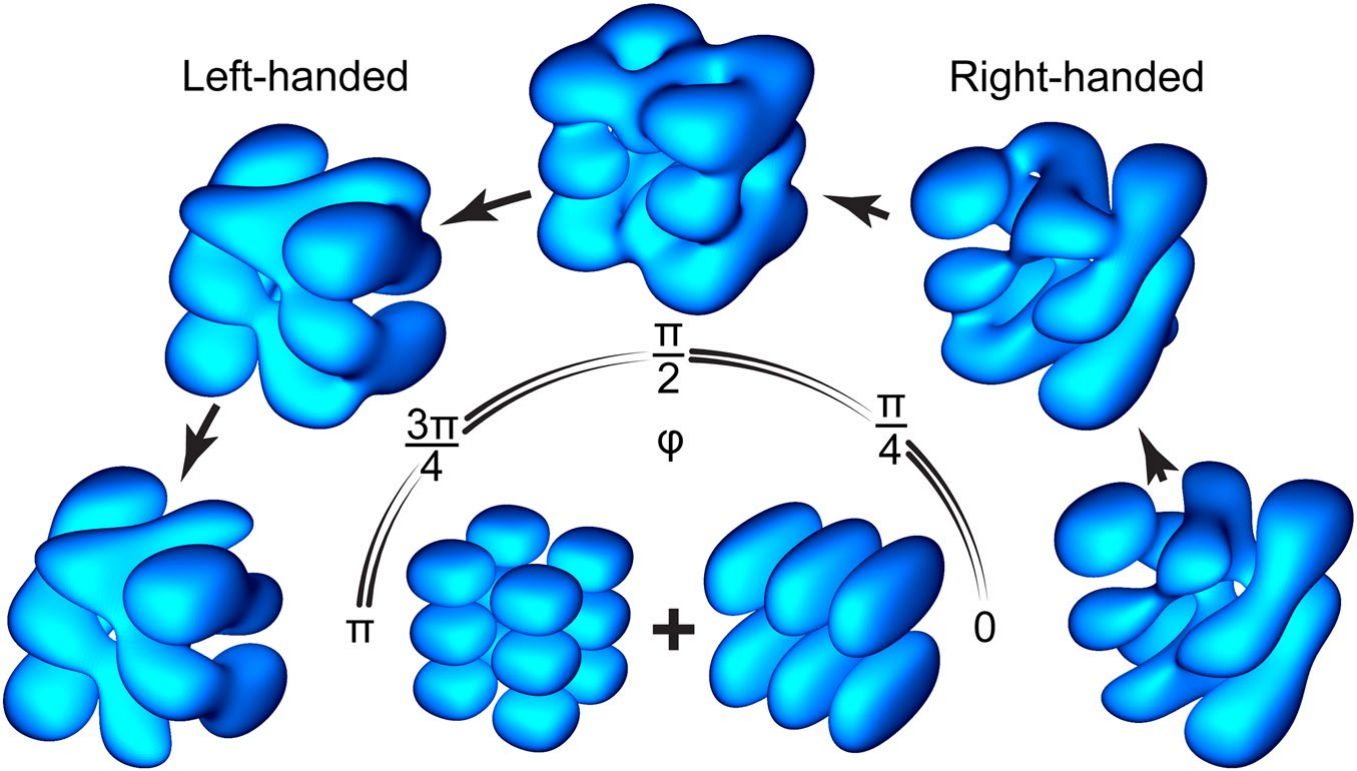
Transformation of probability density |〈r| *f* ^(+)^〉|^2^ with phase *φ* of interaction coupling a pair of resonant nanocuboid states |223〉 and |312〉.

